# Total, Added, and Free Sugars: Are Restrictive Guidelines Science-Based or Achievable?

**DOI:** 10.3390/nu7042866

**Published:** 2015-04-15

**Authors:** Jennifer Erickson, Joanne Slavin

**Affiliations:** University of Minnesota, 1334 Eckles Ave, St Paul, MN 55108, USA; E-Mail: eric2472@umn.edu

**Keywords:** added sugar, free sugar, sugar recommendations, nutrition facts labels

## Abstract

Sugar consumption, especially added sugars, is under attack. Various government and health authorities have suggested new sugar recommendations and guidelines as low as 5% of total calories from free sugars. Definitions for total sugars, free sugars, and added sugars are not standardized, nor are there accepted nutrient databases for this information. Our objective was to measure total sugars and added sugars in sample meal plans created by the United States Department of Agriculture (USDA) and the Academy of Nutrition and Dietetics (AND). Utilizing the Nutrition Data System for Research (NDSR) nutritional database, results found that plans created by the USDA and AND averaged 5.1% and 3.1% calories from added sugar, 8.7% and 3.1% from free sugar, and 23.3% and 21.1% as total sugars respectively. Compliance with proposed added sugar recommendations would require strict dietary compliance and may not be sustainable for many Americans. Without an accepted definition and equation for calculating added sugar, added sugar recommendations are arbitrary and may reduce intakes of nutrient-rich, recommended foods, such as yogurt, whole grains, and tart fruits including cranberries, cherries, and grapefruit. Added sugars are one part of excess calorie intake; however, compliance with low added sugar recommendations may not be achievable for the general public.

## 1. Introduction

Sugar consumption, especially added sugar, has been indicated as a major cause of several chronic diseases prevalent in America including obesity, heart disease, diabetes and dental caries [1]. Results have been inconsistent in determining if increased added sugar consumption is associated with weight gain and obesity rates. Added sugars contain calories and are low in nutrients. The logic states that if added sugar consumption increases without compensation from other calorie sources, calorie intake will also increase which can result in weight gain [1].

Debate continues on whether added sugars play any unique role in obesity. Bray and Popkin [2] argue that sugar-sweetened beverages are associated with several health problems and have effects beyond the calories they add to the diet. In contrast, Kahn and Sievenpiper [3] argue that there is no clear or convincing evidence that any dietary or added sugar has a unique or detrimental impact relative to any other source of calories on the development of obesity or diabetes. Added sugars provide 4 kcal/g just like any other digestible carbohydrate and are no more likely to cause weight gain than any other calorie source [4]. Unlike sodium or dietary fiber that have clear links to health outcomes, added sugar intake is not uniquely linked to negative health outcomes or chronic diseases.

Percent total energy from added sugar has decreased over the past fifteen years, returning to a value close to the typical American diet in the late 1970s [1,5]. Unfortunately, the rate of obesity has not followed the same downward trend, although rates have leveled off among most demographic subgroups. Confusion on definitions for sugars, including total, added, and free will continue to challenge the research community. Additionally, recommendations to consume less than 10% of calories from added sugars, as suggested in recent reports will be impossible to adopt without agreement on definitions and accepted measures for total sugars, added sugars, and free sugars. Also, if these recommendations are not consistent with accepted dietary recommendations and food patterns, it will be impossible for government agencies and consumers to plan and consume diets that are within accepted guidelines for sugar intake.

### 1.1. Sugar

The Food and Drug Administration (FDA) defines sugars as, “the sum of all free mono and disaccharides” which would include glucose, fructose, galactose, lactose and sucrose and maltose [3]. Sugars can be found naturally in foods, including fruits and dairy products, in addition to those sugars that are added to foods during processing. No recommendations currently exist regarding total sugar intake in the United States. There is not sufficient evidence to set a quantitative value as a recommended intake or limit for total sugar intake at this time [3].

### 1.2. Added Sugar

Added sugars are sugars that are not naturally found in the food product and are added during the production of the food. Since USDA developed the added sugars definition, the added sugars term has been used in in the scientific literature. The 2000 Dietary Guidelines for Americans used the term to aid consumers in identifying beverages and foods that are high in added sugars. Although added sugars are not chemically different from naturally occurring sugars, many foods and beverages that are major sources of added sugars have lower micronutrient densities compared with foods and beverages that are major sources of naturally occurring sugars. Currently, U.S. food labels contain information on total sugars per serving, but do not distinguish between sugars naturally present in foods and added sugars.

Calories from added sugars account for approximately 13% of total calories in American adults and 16% in adolescents, according to National Health and Nutrition Examination Survey (NHANES) data from 2005 to 2010 [5]. As published by NHANES, the top sources of added sugar in Americans are sugar-sweetened beverages, desserts, sweetened fruit and candy [6].

Added sugar has been defined by the FDA as, “sugars and syrups that are added to foods during processing or preparation” excluding sugars naturally found in foods, such as fruits or dairy products [7]. Other organizations and nutrient databases define added sugars slightly differently, resulting in a range of values. For example, in the FDA’s proposed revisions to the Nutrition Facts Label, fruit juice concentrates are to be considered as added sugar, while the USDA does not consider any form of fruit juice as added sugar [6,7]. The World Health Organization (WHO) uses the term “free sugar” rather than “added sugar” in their sugar recommendations. Free sugar includes added sugars as well as sugars naturally present in fruit juices as well as fruit juice concentrates [8]. Without a universal and explicit definition, nutrition databases may use different equations to calculate added sugar resulting in a range of values, which could be very confusing to consumers.

### 1.3. Recommendations for Added Sugar Consumption

The range of recommendations for added sugar consumption for Americans is broad and has trended down in recent years, as shown in Table 1. The rationale for establishing added sugar recommendations appears aimed at reducing the total calories in foods high in added sugars, driven by a belief that these food types contribute empty calories, with low nutrient density. Overconsumption of foods high in added sugars therefore may replace other, more nutrient dense foods, and result in nutrient deficiencies or overconsumption of calories [9]. The 2010 Dietary Guidelines for Americans (DGA) suggests that a diet containing more than 15% total calories from solid fats and added sugars cannot comply with a healthy diet within recommended calorie levels [6]. The most recent recommendation was drafted by the World Health Organization (WHO) and released in March 2014, proposing a conditional recommendation of no more than 5% of total energy intake contributing by free sugars [8].

**Table 1:** Current and proposed recommendations for added sugar intake for Americans [8,9].

Institution	Recommendation
Institute of Medicine (2002)	<25% total energy intake from added sugars
World Health Organization current recommendation (2003)	<10% total energy intake from free sugars
American Heart Association (2009)	No more than half of discretionary calorie intake from added sugars. 100 calories for females, 150 calories for males.
USDA Dietary Guidelines for Americans (2010)	5%–15% total energy from solid fats and added sugars
World Health Organization conditional recommendation (2015)	Aim for <5% total energy intake from free sugars

Most public health opinion leaders agree that added sugar should be consumed at a minimum to prevent excess intake of calories while taking in all necessary nutrients. The purpose of this paper is to determine the percent of total energy from total sugars and added sugars using meal plans designed by organizations responsible for dietary guidance. The objective is to determine if it is possible to meet the current and proposed recommendations following a recommended food pattern. Published meal plans from the USDA and the Academy of Nutrition and Dietetics were examined using current nutrient analysis software that calculates added sugar contents in food items.

## 2. Experimental Section

### 2.1. Added Sugar Databases

Very few existing nutrient databases have information regarding added sugar contents in food products. Nutrition Data System for Research (NDSR) version 2014 includes added sugar estimations in its nutrient analysis and also includes food mixtures and commercial products so that it why it was chosen as the database for this work. NDSR contains a comprehensive nutrient database comprised of more than 18,000 food products including brand names. NDSR calculates added sugars in two separate ways: Added sugars by available carbohydrate, as well as added sugars by total sugars. Added sugars by available carbohydrates include all carbohydrates added as a caloric sweetener into the product as added sugars. This includes monosaccharides, disaccharides and polysaccharides. Added sugars by total sugars only includes monosaccharides and disaccharides that were added as caloric sweeteners as added sugars [10]. The method by which NDSR calculates the added sugar amounts is not public knowledge, however it likely leverages an equation that utilizes the ingredient list to reverse engineer the approximate amount of added sugar present in the product.

The USDA developed a database for providing estimated added sugar values in food products. This database utilizes the ingredient list and provided total sugar amounts to calculate the approximate added sugar value [9]. This database is no longer accessible to the general population and was removed from the USDA website in 2012 due to the constant changes in the formulation of food products [11].

Table 2 provides a sample of common foods with the assigned added sugar values as determined by the NDSR software and the last version of the USDA added sugar database (2009–2010). Total sugar- USDA data was obtained from the National Nutrient Database for Standard Reference [12,13]. We also attempted to include information from the most recent (2011–2012) USDA database, the Food Patterns Equivalent Database (FPED), although it was difficult to use the information for recipes and complicated foodstuffs.

**Table 2:** Approximate total and added sugar content of selected foods.

Food	Common Serving Size	Serving Size (g)	Total Sugars	Added Sugar—by Total Sugars (g)	Added Sugars—by Available Carbohydrate (g)	Total Sugars (g)—USDA	Added Sugars (g)—USDA	USDA FPED 2011–2012
*Grains*								
Bagel, plain	1 medium—3.5'–4"	105	5.3	0.0	0.0	8.9	5.1	5.1
Bread, white	1 medium slice	25	1.3	1.0	1.0	1.4	1.0	1.0
Bread, whole wheat	1 medium slice	28	2.9	1.4	1.9	1.2	1.5	1.2
Chocolate cake, frosted, prepared from mix	2" × 2" slice	40	23.5	23.4	26.4	16.0	14.9	15.6
Cereal, corn flakes	1 cup	28	2.9	2.7	2.9	2.7	2.6	2.3
Cereal, corn flakes, frosted	1 cup	40	15.5	15.2	17.6	14.2	15.2	13.9
Brownie	2" × 2" slice	43	17.5	17.2	17.3	15.7	15.7	15.4
Cookie, chocolate sandwich	1 cookie	12	4.9	4.1	4.1	4.9	4.8	4.8
Crackers, graham, plain	1 large rectangle	14	4.4	3.6	3.8	3.5	4.3	4.3
Doughnut, cake-type, frosted	3.25" diameter	67	21.4	20.8	21.1	17.9	15.0	16.6
Muffin, blueberry	3" diameter	113	16.7	12.6	12.7	35.6	29.1	32.7
Pie, apple, lattice crust	1/8 of 9" pie	162	26.5	18.7	18.7	25.4	17.4	17.4
Popcorn, caramel-coated	1 cup	35	19.7	19.4	23.0	18.6	18.3	18.2
*Fruit*								
Applesauce, sweetened	1 cup	246	36.1	21.2	21.3	36.1	10.3	13.0
Applesauce, unsweetened	1 cup	244	22.9	0.0	0.0	22.9	0.0	0.0
Blueberries, frozen, sweetened	1 cup	230	45.4	25.3	25.3	45.4	25.8	25.9
Cranberries, dried, sweetened	1/2 cup	60	39.0	39.0	39.1	39.4	32.6	32.6
Peaches, heavy syrup, canned	1 cup	262	48.8	33.5	33.5	48.8	36.2	36.2
Peaches, in juice, canned	1 cup	248	25.5	0.0	0.0	25.5	0.0	0.0
Raisins	1/2 cup	72.5	42.9	0.0	0.0	42.9	0.0	0.0
*Dairy*								
Cream substitute, liquid	1/2 oz	14	0.7	0.7	1.9	4.6	1.6	1.6
Milk, plain 1% milk fat	1 cup, 8 fl oz	244	12.7	0.0	0.0	12.7	0.0	0.0
Milk, chocolate, low-fat	1 cup, 8 fl oz	250	24.9	13.4	13.4	24.9	16.6	11.9
Yogurt, plain, low-fat	1 cup	245	17.2	0.0	0.0	17.3	0.0	0.0
Yogurt, vanilla, low-fat	1 cup	245	33.8	15.0	15.0	33.8	16.7	16.7
Yogurt, fruit-flavored, low-fat	1 cup	245	40.2	10.2	30.8	45.7	29.5	29.5
Ice cream, vanilla	1/2 cup	66	14.0	8.0	12.1	14.0	12.0	11.6
*Sugar-sweetened beverages*								
Cola, regular	1 can, 12 fl oz	368	33.0	33.0	35.2	33.0	33.1	39.6
Soda, lemon-lime	1 can, 12 fl oz	370	33.2	33.2	37.5	38.3	33.3	33.3
Fruit punch	8 fl oz	227	25.6	25.6	26.7	25.9	23.6	21.6
Sports drink, all flavors	8 fl oz	227	11.9	11.9	14.6	11.9	11.9	11.9
*Other*								
Candies, hard	1 piece	3	1.9	1.9	2.8	1.9	1.9	1.9
Gelatin	3.25 oz	92	12.5	12.5	13.1	12.4	12.4	12.4
Milk chocolate	1.55oz bar	43	22.1	20.2	20.2	22.1	18.9	18.0
Peanut butter, regular	2 Tbsp	32	3.0	1.6	1.6	3.4	1.7	1.7
Jelly	1 Tbsp	19	9.2	8.2	12.3	9.7	9.7	9.1

Total and added sugar values calculated from USDA Added Sugar database, USDA Food Pattern Equivalents Database (FPED) and NDSR database.

### 2.2. Nutrient Analysis

Meal plans created by the USDA and Academy of Nutrition and Dietetics (AND) were analyzed utilizing a nutrient database to assess total and added sugar content of daily consumption. Both meal plans were accessible online and utilized a multiday meal plan approach to help consumers plan and consume the recommended diet and modeling day-to-day variation. The meal plans were analyzed using Nutrition Data System for Research software version 2014, developed by the Nutrition Coordinating Center (NCC), University of Minnesota, Minneapolis, MN. The first seven days were selected from a “Sample 2-Week Menu” made available via USDA’s ChooseMyPlate.gov website [14]. Corresponding food items were selected from the NDSR database. Some menu items referenced recipes that were made available from a corresponding cookbook [15]. These recipes were entered into the NDSR database allowing for the analysis of the precise recipe that was recommended in the menu. The Academy of Nutrition and Dietetics provides sample menus for client education through the Nutrition Care Manual^®^. The “1800-Calorie 5-Day Menu” was used as a sample menu and analyzed [16]. Food from each of the five days was entered into and analyzed by NDSR.

Each day was analyzed individually populating total energy, total carbohydrate, total sugars, added sugars-by total sugars, and added sugars-by available carbohydrate. Percent of total energy from total sugars and percent of total energy from added sugars were calculated by multiplying the grams of total sugar and added sugar, respectively, by four and dividing by the total number of calories. Free sugars were estimated by adding the grams of sugar from fruit juice used as beverages, to grams of added sugar-by total sugars. Percent of total energy from free sugars was estimated by multiplying the calculated free sugars by four and dividing by total calories.

## 3. Results

**Figure 1:**
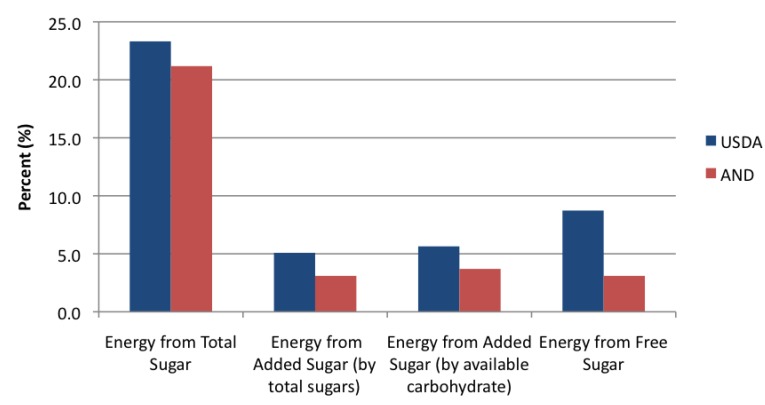
Average percent energy from total, added, and free sugar from sample menus designed by the United States Department of Agriculture (USDA) and the Academy of Nutrition and Dietetics (AND).

**Figure 2:**
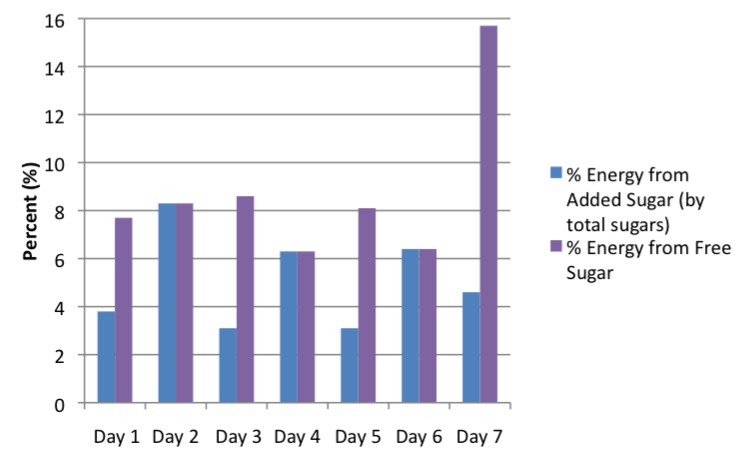
Percent energy from added sugar *versus* free sugar in the USDA sample menu by day.

The “Sample 2-Week Menu” made available by the USDA provides on average 23.3% energy from total sugar and 5.1% energy from added sugars-by total sugars. The “1800-Calorie 5-Day Menu” designed by AND provides on average 21.2% total energy from sugars and 3.1% energy from added sugar-by total sugar (Figure 1). It is possible to consume less than 5% of total calories from added sugar with strict compliance of these provided meal plans. However, the WHO’s proposed recommendation of less than 5% of total energy from free sugars, includes fruit juices. The menu designed by the USDA uses juice as a beverage on several days, and the substantial difference between added sugar and free sugar is depicted in Figure 2. On average, the USDA sample menu provides 8.7% energy from free sugars, which is more than the WHO’s recommended <5% of total energy. The AND meal plan does not provide juice as a beverage option, so the AND meal plan remains below the WHO recommendations at 3.1% total energy from free sugars.

## 4. Discussion

### 4.1. Meeting Proposed Recommendations

It is possible to meet the stringent free sugar recommendation proposed by the WHO for consumers following a diet similar to the AND meal plan. The AND recommended daily menu provides 8 one-ounce servings of lean protein, 14 servings of carbohydrates (includes grains, dairy products and fruit), at least 3 servings of vegetables and 5 servings of fat. Besides fruit, a sweet snack is only provided twice throughout the 5-day menu. The listed desserts are sugar free gelatin and sugar free pudding. According to NHANES 2003–2004 data, the median vegetable intake (excluding fried potatoes) in adult men is less than 1.5 cups per day and the median fruit consumption was just over a half cup daily (0.61 cups). The most common fruit consumed by Americans was orange juice, which would contribute to free sugar consumption [17]. Additionally, about 24% of Americans consume at least one sugar sweetened beverage each day [18]. To comply with the AND meal plan, most Americans would have to at least double their fruit and vegetable consumption and eliminate all juice and sugar sweetened beverages from their daily routine. Following this meal plan would be very challenging for the average American as it is drastically different from the typical diet.

A recommendation of less than five percent free sugars or added sugars requires strict compliance to a diet, similar to the diets analyzed in this paper, and does not allow for indulgences. An individual with a 2000 calorie energy requirement could consume all necessary daily nutrients and consume a 150 calorie can of soda within the 2000 calorie budget. The soda alone results in 7.5% energy from added sugar. It would seem doubtful for the American population to sustain a diet that does not allow for even small treats in their daily routine.

The meal plan provided by AND utilizes sugar-free products and artificial sweeteners to achieve a low quantity of added sugars. Not all consumers enjoy the taste of artificial sweeteners and while approved as safe by many regulatory bodies globally, may not be comfortable with the safety of synthetically derived food ingredients [19]. Survey results published in 2006 found a majority of consumers, 81%, prefer the taste of sugar compared to artificial sweeteners. Additionally, 64% of those surveyed were concerned regarding the potential health risks of consuming artificial sweeteners [20]. Achieving the recommended free sugar recommendation would not be possible if consumers are not willing to consume the sugar-free products or abstain from sweet treats.

Both of these analyzed menus provide an example of how to achieve low dietary intakes of added sugars and certainly represent a healthy diet to strive for. Following either of these diets would be an improvement from the current average American added sugar intake of 13% of total energy [6]. Because the menu designed by the USDA to comply with the MyPlate food guide exceeded the WHO’s recommendation for free sugars, the WHO’s proposed recommendation appears unrealistic at this time. Additionally, there is some question if consumers would possess the resources to assess their added sugar intake and comply with recommendations.

### 4.2. Measuring Added Sugar

As noted earlier, most nutrient databases do not contain added sugar values. For those databases that do have the information, the values are not always consistent. Table 2 compares the added sugar contents in the same food products between the USDA and NDSR databases. Some variation is expected, as the formulation of the food product identified may not be identical between databases. Additionally, the definition of “added sugar” and the method of calculations may differ between databases. For example, in looking at sweetened applesauce, one cup of applesauce has identical total sugar values between NDSR and the USDA database (36.09 g). However, NDSR identifies 21.2 g as added sugars, while the USDA database only classifies 10.3 g sugar as added sugar. These types of disparities can make it challenging for consumers to understand and comply with recommendations. Without a standard definition and database to identify added sugar contents in food products, it is nearly impossible for dietitians and consumers to assess compliance.

As previously stated, NDSR distinguishes between added sugar- by total sugars and added sugar—by available carbohydrates based on the chemical structure of the carbohydrates in caloric sweeteners. Caloric sweeteners considered in the equation by NDSR include: sucrose, brown sugar, powdered sugar, honey, molasses, pancake syrup, corn syrup, high fructose corn syrup (HFCS), invert sugar, invert syrup, malt extract, malt syrup, fructose, glucose, galactose and lactose. Added sugar—by available carbohydrates includes all carbohydrates in the caloric sweeteners, not just mono and disaccharides [10].In some foods, added sugar-by available carbohydrate content can be higher than the total sugar content in the product because oligosaccharides and polysaccharides do not contribute to total sugar, but would contribute to added sugar—by available carbohydrate. Corn syrup is likely the largest contributor to the increased value of added sugars—by available carbohydrate. Starches from corn are chemically broken down into sugars in corn syrup. However, not all of the starch is converted into glucose and fructose. Some oligosaccharides and polysaccharides remain in the syrups and would count as added sugars by available carbohydrate [21]. Foods in Table 2 with considerable variation between added sugar contents include caramel-coated popcorn, ice cream, fruit flavored yogurt, soda, sports drinks and jelly. All of these products are typically manufactured with some inclusion of corn syrup.

Because the FDA’s definition of sugar only includes monosaccharides and disaccharides, it is likely that the category of added sugars- by available carbohydrate will not be used in legislation. But this discrepancy does create another point of confusion for consumers.

### 4.3. FDA Proposed Addition of “Added Sugar” to Food Labels

The FDA proposed changes to the Nutrition Facts and Supplement Facts label in March of 2014. The purpose of updating the Nutrition Facts label is to assist consumers in choosing foods that will help them to comply with a healthy diet and meet dietary recommendations. The FDA proposed many changes, including the addition of the category of “Added Sugars” listed beneath “Sugars” or “Total Sugars”. The proposal from the FDA defines added sugars as sugars and syrups added during the food manufacturing process. Fruit juice concentrates are considered added sugars, while fruit juice is not considered to be added sugar [6].

The addition of an “added sugars” category on the food label would provide a tool for consumers to assess compliance with added sugar recommendations and compare food products. Added sugar values in nutrient databases would be more available, and ideally, a standardized equation would be used to calculate added sugar. The FDA hopes this addition will help consumers in making healthier choices and will cause food manufacturers to alter product formulations to decrease the amount of added sugars [6].

Added sugar is chemically identical to sugars naturally found in the foods, therefore, food companies will have to utilize calculations to determine the amount of added sugar included in each food product. The FDA document anticipates requiring manufacturers to keep records verifying the amount of added sugars present in each step of processing for each food product [6]. Without a method to analytically test for added sugars, the food manufacturers would likely have to disclose their product formulations or other proprietary information to the FDA to confirm that the provided added sugar value is accurate. Food manufacturers would be resistant to provide this information, recognizing that the proprietary knowledge represented by their specific recipes give them an important edge over the competition.

The proposed food labels display added sugars in grams per serving. However, the majority of the added sugar recommendations are made in terms of percentage of total calories. In order to check personal compliance with added sugar recommendations, consumers would have to convert total grams of added sugar to percent of total calories from added sugars. This begs the question; will consumers know what to do with the information? The FDA has considered presenting the added sugars value in calories from added sugars rather than grams presumably to help consumers better relate this value to the recommendations made by the various organizations [6]. This format would still require some calculations and conversions and may cause confusion due to the difference in units between sugars and added sugars. Both methods of presentation have clear limitations. The goal of the new Nutrition Facts label is to help consumers make better-informed decisions when purchasing food products, but the addition of added sugars on the label may be more confusing to the consumer.

A study conducted by the Turner Research Network, asked consumers to look at food labels and determine the total amount of sugar in the product. According to preliminary data, participants who were shown a label that only had “sugars” listed, 92% were able to accurately identify the total amount of sugar. When “added sugars” was added below the “sugars” category, only 55% accurately identified the amount of total sugar. Notably, 52% of participants thought that the “added sugars” were in addition to the total amount of sugars listed on the label [22]. The inclusion of the “added sugars” category appears to make interpreting the Nutrition Facts label more difficult and may actually be doing the opposite of what the FDA had hoped to do.

## 5. Conclusions

At this point in time, it would be difficult to measure compliance with added sugar recommendations. There is no universal definition for “added sugars”, consumers do not have an easily accessible method to calculate added sugar consumption, and various organizations utilize different units of measurements for their recommendations. Newly proposed recommendations provided by the WHO encourage limiting added sugar intake to less than 5% total energy intake from free sugars. A meal plan provided by the USDA, following MyPlate dietary guidelines, was unable to achieve such strict recommendations. Although a menu developed by the AND demonstrates that it is possible to achieve the WHO recommendations, it would be very difficult for the average American to follow such a restrictive diet for an extended period with no allowance for any indulgences. While it is important to minimize discretionary calories, it is also important to follow a diet that is sustainable for the individual. The proposed free sugar recommendation from the WHO is likely too restrictive and unachievable for most Americans.
